# Does Imagery Ability Matter for the Relationship Between Temperament and Self-Confidence in Team and Individual Sport Disciplines?

**DOI:** 10.3389/fpsyg.2022.893457

**Published:** 2022-06-29

**Authors:** Dagmara Budnik-Przybylska, Izabela Huzarska, Karol Karasiewicz

**Affiliations:** ^1^Department of Sport Psychology, Faculty of Social Science, Institute of Psychology, University of Gdańsk, Gdańsk, Poland; ^2^Institute of Physical Culture Studies, Medical College of Rzeszów University, University of Rzeszów, Rzeszów, Poland; ^3^Institute of Psychology, University of Szczecin, Szczecin, Poland

**Keywords:** general imagery, self-confidence, temperamental axes, emotionality, sport

## Abstract

The main purpose of our study was to investigate the relationship among temperamental traits, a general tendency to use imagery, and self-confidence in sport. The specific aim was to verify if general imagery mediates the relationship between temperament and self-confidence in sport, but also with respect to individual and team sport disciplines. The study involved 144 athletes aged 19–25 years (75 men and 69 women) with different lengths of training (from 1 month to 18 years) and presented different sports levels: international (*n* = 12), national (*n* = 46), and recreational (*n* = 86). They also represented individual (*n* = 73) and team (*n* = 68) sports. The Imagination in Sport Questionnaire (ISQ), Trait Sports Confidence Inventory (TSCI-PL), and Temperament Questionnaire (EAS) were all completed by the participants. Results indicate that self-confidence is explained in about 13% by temperament and general imagery, but only general imagery (*b* = 0.22; *p* < 0.05) and negative axes of the temperament—emotionality (*b* = –0.26; *p* < 0.05). The test of the indirect effect of the negative axes of the temperament on self-confidence through general imagery, conducted by Preacher and Hayes bootstrapping procedure, revealed significant mediation [*b* = 0.37; 95% *CI* = (0.09; 0.68); *R*^2^_Med_ = 0.46] suggesting that about 46% of variance explained in self-confidence by emotionality is related to general imagery. The other 3% of variance explained in self-confidence by the positive temperament axes is related to general imagery, however, it was insignificant. The general imagery explains the relationship between emotionality and self-confidence only in individual sports athletes.

## Introduction

Nowadays, an increasingly important role in the work of psychologists with athletes is to work on the sphere of imagery, so it is important to deepen the knowledge of this area of psychology to use it in daily training work (Morris et al., 2005; Cumming and Eaves, 2018). Imagery can be divided into ability and use, where *imagery ability* is the “individual capability of forming vivid, controllable images, and retaining them for sufficient time to effect the desired rehearsal” (Morris et al., 2005), conversely the *use of imagery*, i.e., “use of imagery to achieve a variety of cognitive, behavioral, and affective changes” (Murphy and Martin, 2002, p. 418). Use is a part of mental training and imagery training. It includes the process of internal or external imagery of motor exercises, movement, and features of a specific discipline (Dietrich, 2008; Yadolahzadeh, 2020). One of the often-used model in imagery training connected to imagery use is the PETTLEP model (Holmes and Collins, 2001). On the other hand, it is important to identify the role of personality factors to be able to relate them to imagery abilities (Cumming and Eaves, 2018).

As Cumming and Williams (2013) showed in the revised applied model of deliberate imagery use (RAMDIU), the component “who” embraces individual characteristics, such as personality and temperament. For example, dimensions of individual empathy influence on imagery is sport. People with high other-oriented empathy are more likely to use this technique naturally without requiring an incentive. On the other hand, being focused on experiencing the negative emotions of others (personal distress) is associated marginally with imagery (Budnik-Przybylska et al., 2021).

Previous research in a group of ballet dancers revealed that some personality dimensions, primarily openness to experience and conscientiousness, have an impact on imagery ability (Budnik-Przybylska et al., 2019). Moreover, other personality traits, such as neuroticism correlate negatively while extraversion correlates positively with imagery in sports (Budnik-Przybylska et al., 2018). Jeunet et al. (2015) showed that personality traits, such as tension, abstractness, and self-reliance measured by sixteen personality (16PF) factors have a significant association with mental imagery using brain-computer interfaces. Imagery ability was best predicted by a combination of vividness of visual imagery with particular factors: orderliness, autonomy, and emotional stability (Leeuwis et al., 2021).

One of the most commonly used theories of temperament is the emotionality, activity, shyness, and sociability (EAS) model (Buss and Plomin, 1984) which connects inherited personality traits to emotionality, activity, and sociability. Temperament, as genetically determined and revealed in early life, constitutes the foundation for personality development (Buss and Plomin, 1984). Temperamental traits also predict imagery use (Budnik-Przybylska and Kuchta, 2020), mainly emotionality as a negative predictor. In addition, some other temperamental characteristics have an association with motor imagery skills (Zapała et al., 2020) in which there were significant correlations between information transfer rate (ITR) performance and endurance, but also perseveration scores. The study of temperamental correlates measured by the Formal Characteristic of Behavior-Temperament Inventory (FCB—TI, Strelau and Zawadzki, 1995) of the ability to use imagery using the Imagination in Sport Questionnaire (ISQ; Budnik-Przybylska, 2014) showed that activity as temperamental traits positively correlates with the most of the ISQ subscales, while emotional reactivity (tendency to react intensively to emotion generating stimuli)—negatively. Perseverativity [tendency to continue and to repeat behavior after cessation of stimuli (situations)] on the other hand, correlated positively with the scale of feeling physiological sensations during imagery (Budnik-Przybylska et al., 2018). Continuing the revised applied model of deliberate imagery use (RAMDIU, Cumming and Williams, 2013), there can be an examined function (the why component) of the imagery. Self-confidence, which consists of expectations and awareness of athletes’ resources contributes to the creation of positive emotions, increased the level of commitment, and influences on sports performance (Plakona et al., 2014), and athletes’ performance can also be boosted by confidence (Vealey, 2009). As the previous research of athletes based on the aforementioned model confirms, imagery could be used to work on self-confidence (Cumming and Williams, 2013; Munroe-Chandler et al., 2013; Cumming and Eaves, 2018). On the other hand, by working on reinforcement of confidence, we can strengthen our imagery skills (Williams et al., 2021). The imagery ability also has a positive influence to lower anxiety and stress feeling (Yadolahzadeh, 2020). Although, as Williams et al. (2021) showed, confidence is related to mastery imagery, but not as a mediator between imagery and the level of more positive interpretations of somatic and cognitive anxiety.

### Temperament Versus Personality and Self-Confidence

Athletes have various personal features that may have an impact on different factors, which are directly or indirectly linked to self-confidence. Temperamental traits are diversifying the level of stress, which showed in the study conducted on a group of cyclists (Samełko and Tomaszewski, 2020). Some personal traits, such as openness and emotional reactivity, have a limited effect on athletes’ performance of postural balance (Wojciechowska-Maszkowska et al., 2020).

Personality factors can have a role as a limited indicator of confidence. Self-confidence can be significantly predicted by high scores in neuroticism, conscientiousness, and openness (Ong and Harwood, 2018). Neuroticism has negative associations with self-confidence (Matsumoto et al., 2000; Balyan et al., 2016). Additionally, research based on Brazilian athletes who train individual or team sports showed that self-confidence has a negative relationship with cognitive anxiety (Fernandes et al., 2013). In addition, the more confident the person is, the lower the level of cognitive, somatic, and state anxiety (Kolayiş and Sari, 2011).

Self-confidence correlates positively with narcissism in physical education students before the archery exam (Dal, 2018). Moreover, self-report confidence is significantly related to extraversion and emotional stability (Burns et al., 2016), resilience (Brooks and Goldstein, 2003; Westmattelmann et al., 2021), optimism (Westmattelmann et al., 2021), and conscientiousness (Matsumoto et al., 2000).

### Differences Between Individual and Team Sports Athletes in Imagery

Reflections in the context of imagery ability can be carried out with the inclusion of sport specifics. There are differences in the imagery abilities of athletes who train for individual sports and those who train for team sports. Athlete’s sport-oriented imagery skills vary depending on the type of sport, individual, or team, they practice. Most of the diversity of images was observed in representatives of individual sports. These athletes achieved higher results in the vividness of visual imagery ability and also in external visual imagery than athletes from team sports (Di Corrado et al., 2019). Additionally, there are some differences in general imagery of using motivational imagery functions in individual or team athletes. Athletes from individual sports had significantly higher results in motivation general-arousal, motivation general-mastery, and kinesthetic imagery ability (Peltomäki, 2014).

Based on the above-mentioned literature, we decided to explore more in-depth the relationship among temperamental traits, general tendency to use imagery, and self-confidence in sport, but also specifically in individual and team disciplines. We expected that general imagery could mediate the relationship between personality traits and self-confidence. Therefore, we constructed the model presenting those linkages and formulated the following hypotheses.

H1. General imagery measured by ISQ is the mediator between temperament and self-confidence in athletes’ population.

H2. The sport discipline (individual vs. team sports) is moderating the indirect effect of temperament on self-confidence through general imagery.

## Participants

The participants taking part in the study were students of the Academy of Physical Education and Sport in Gdansk. There were a total of 144 people aged 19–25 years, 75 men and 69 women. The athletes’ training experience ranged from 1 month to 18 years. Among the subjects, 12 athletes train at the international level, 46 at the national level, and 86 respondents train at a recreational level. The examples of disciplines that the examined athletes train on a daily basis are: team sports: volleyball, soccer, basketball, and handball; individual: swimming, combat sports, sailing, rowing, and tennis.

All subjects gave their informed consent for inclusion before their participation in the study. The study was conducted in accordance with the Declaration of Helsinki, and the Ethics Committee of the University of Gdańsk approved the protocol (11/2015) before the commencement of the study.

## Materials and Methods

### Imagery

The ISQ (Budnik-Przybylska, 2014) is a multidimensional 51-item measurement tool with seven subscales, i.e., physiological feelings (noticeable changes in body functioning), modalities (use of senses besides the visual sense), ease/control (ease and control of the imagined scene), perspective (juggling of different perspectives of the imagined scene), affirmations (positive attitude during competition), visual (visual sense), and general (general tendency to use imagery). The participants imagined themselves before a start in a high-level competition for 60 s and after this task responded to the 51 items, assessing the different aspects of the image on a scale from 1 (not at all) to 5 (completely so). All subscales (except one that was named “general”) were related to the imagined situation, i.e., situational imagery. The “general” subscale consisted of six questions and was developed separately to assess the general tendency to use imagery, i.e., general imagery. The ISQ demonstrates good stability (test–retest reliability ranged from *r* = 0.55 to *r* = 0.74) over a 3-week period and sound internal consistency (indicated by Cronbach’s alpha, which ranged from 0.64 to 0.79). A confirmatory factor analysis indicated acceptable model fit indices for the ISQ’s seven-factor structure normed chi-square (NC) = 2,416.63, degree of freedom (df) = 1,203, Goodness of fit index (GFI) = 0.944, AGFI (Adjusted Goodness of Fit index) = 0.944, root mean square error of approximation (RMSEA) = 0.056. In our study, we used only the last scale-general imagery as general tendency to use imagery. Situational imagery is connected to the specific competition situation, therefore we decided that it should not be the mediator between temperament and sport self-confidence. Below we present examples of items from the general imagery subscale: Do you generally create ideas easily?; Do you imagine the events waiting for you?; Do you use your imagination in everyday life?; and Do positive events dominate in your imagination?

### Self-Confidence

The Trait Sports Confidence Inventory (TSCI-PL) (Robin S. Vealey, Polish adaptation: Gazdowska et al., 2017) is based on the original TSCI created by Robin S. Valey. TSCI-PL was adapted to use in Poland by Z. Gazdowska, D. Parzelski. It consists of 13 self-assessment items. The respondent is asked to answer the given statements on a nine-point Likert scale (1: low, 9: high), where the participants are asked to state how self-confident they feel in sport-specific situations, compared with the most self-confident athlete they know (i.e., “Compare your confidence in your ability to perform under pressure to the most confident athlete you know”). The reliability of the TSCI-PL is relatively high (0.94) and the individual items ranged between 0.64 (item 13) and 0.76 (item 8). A confirmatory factor analysis indicated acceptable model fit indices for the single factor structure of TSCI-PL. The RMSEA = 0.076, GFI = 0.923, Bentler-Bonett Normed Fit Index (NFI) = 0.923, and Comparative Fit Index (CFI) = 0.954 exceed the reasonable fit of 0.9.

### Temperament

The EAS Temperament Questionnaire (Arnold H. Buss, Robert Plomin, Polish adaptation: Oniszczenko, 1997) checks inherited temperament traits. The adult version of the questionnaire is self-descriptive, consists of 20 items, and requires a five-point response scale on the truthfulness of the statements. The tool has five scales concerning: Dissatisfaction—the tendency to react with anxiety in an easy and strong way, a high score means intense reactions and difficulties in controlling (e.g., crying and screaming), Fear—indicates a tendency to react or respond on stimuli with anxiety, tension, and expectation of the negative event, Anger—reacting with anger expressed both in facial expression, motor, and cognitive signs, Activity—physical energy, the components of which are speed and vigor, and Sociability—the desire and need to seek contact with others and to avoid loneliness. The first three subscales are called emotionality. The theoretical basis of the tool is the Genetic Theory of Temperament by A. H. Buss and R. Plomin, where temperament is understood as a set of inherited personality traits. The reliability of the Polish version of EAS ranged between 0.57 for sociability to 0.74 for dissatisfaction. The criterion validity of the measure was confirmed. In our study, we used the term negative axes for emotionality and positive axes for activity and sociability.

### Procedure

The athletes were invited to participate in the study by the academic teachers in charge. After agreeing to participate in the study, they completed all the above tests (ISQ, EAS, and TSCI-PL). The questionnaires were distributed in random order. There was no time limit within which the athletes had to complete the above tests.

#### Primary Analysis and Data Preparations

The Mauchly Test of Sphericity and the Shapiro–Wilk Test of unidimensional normality have been chosen due to their practical use in the Box-Cox transformation (Vélez et al., 2015). To test if multivariate linear models (as linear regression and structural equation modeling [SEM]) assumptions of univariate and multivariate normality are met in collected data, a Shapiro–Wilk normality test (to test univariate normality) and Mauchly’s test of sphericity were performed across all indices of ISQ and EAS scores. In cases of observed significant deviation of univariate normal distribution, the Box-Cox procedure of logarithmic transformation was induced. The results are presented in the Table 1 below.

**TABLE 1:** Descriptive statistics and Shapiro–Wilk’s test of normality in collected dataset.

		Descriptive statistics				Shapiro-Wilk *p*-value	Box-Cox transformation	
		Min	Max	*M*	SD		l	Shapiro-Wilk
ISQ	General	0	30	25.04	3.55	0.000	3.44	0.056
EAS	Dissatisfaction	4	13	10.42	3.28	0.018	0.59	0.068
	Fear	4	18	9.56	2.93	0.022	0.70	0.062
	Anger	4	20	12.12	3.31	0.191	0.93	0.197
	Activity	6	20	13.86	2.93	0.049	0.94	0.059
	Sociability	6	19	13.63	3.04	0.003	1.41	0.071
Self-confidence		13	148	74.63	21.00	0.364	1.05	0.378

In the athletes’ population, scores of tested psychological phenomena appeared to be not normally distributed, probably because of the special use of those measures, which are constructed to measure psychological phenomena in different populations, such as general young students (EAS) or high-level athletes (ISQ). This new use of those measures slightly decreases their validity and reliability. Scores transformed using Box-Cox logarithmic transformation appeared to be normally distributed, i.e., after Box-Cox transformation, results met assumptions of univariate normality and sphericity.

First, we conducted a Pearson correlation between general imagery from ISQ and EAS. Results are presented in Table 2. Analysis revealed that sociability and activity significantly support general imagery, although correlations are moderate, while correlations between fear and dissatisfaction and general imagery are negative. Similarly, temperament corresponds with self-confidence, where fear, anger, and dissatisfaction correlate negatively, while activity and sociability correlate positively with self-confidence. These results suggest that general imagery, as a significant correlate of self-confidence (*r* = 0.26 and *p* < 0.01), could mediate between temperament and self-confidence in a population of athletes.

**TABLE 2:** Correlations among general imagery, the emotionality, activity, shyness, and sociability (EAS) temperament, and self-confidence.

	General imagery	Dissatisfaction	Fear	Anger	Activity	Sociability	Self-confidence
General imagery	1	−0.254**	−0.165*	−0.132	0.262**	0.299**	0.256**
Dissatisfaction	−0.254**	1	0.621**	0.616**	−0.094	−0.474**	−0.284**
Fear	−0.165*	0.621**	1	0.358**	−0.032	−0.295**	−0.317**
Anger	−0.132	0.616**	0.358**	1	0.032	−0.313**	−0.160
Activity	0.262**	−0.094	−0.032	0.032	1	0.155	0.045
Sociability	0.299**	−0.474**	−0.295**	−0.313**	0.155	1	0.182*
Self-confidence	0.256**	−0.284**	−0.317**	−0.160	0.045	0.182*	1

**p < 0.01; *p < 0.05.

## Results

To verify the main hypothesis that general imagery measured by ISQ is a mediator between temperament and self-confidence in the athletes’ population. An SEM procedure was conducted using generalized least squares (GLS) estimation. To obtain indirect effects, standard errors (SEs), and estimate exact their *p*-values. Hayes and Preacher’s (2010) procedure using the non-parametric bootstrap percentile method was conducted.

**FIGURE 1:**
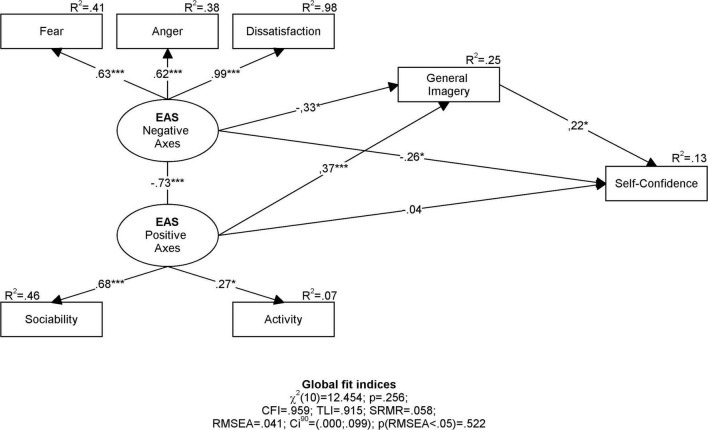
Result diagram of SEM model estimated in athletes sample (*N* = 144). *p < 0.05, **p < 0.01, ***p < 0.001.

Results of the analysis indicate that self-confidence is explained by about 13% by temperament and general imagery, but only imagery (*b* = 0.22; *p* < 0.05), and negative axes of the athletes’ temperament (*b* = –0.26; *p* < 0.05). Test of the indirect effect of the negative axes of the temperament on self-confidence through general imagery conducted by Hayes and Preacher bootstrapping procedure revealed significant mediation [*b* = 0.37; 95% *CI* = (0.09; 0.68); R^2^_Med_ = 0.46] suggesting that about 46% of variance explained in self-confidence by negative temperament axes is related to general imagery. Analogous analysis conducted to test the indirect effect of the positive axes of temperament on self-confidence through general imagery did not reject the null hypothesis of zero indirect effect (non-significant mediation) [*b* = –0.02; 95% *CI* = (–0.37; 36); R^2^_Med_ = 0.03] indicating that general imagery explains only 3% of the self-confidence variance explained by positive temperament axes. The results are shown in the (Figure 1).

The second analysis is related to the hypothesis that characteristics of the sport discipline (individual vs. team sports) are moderating the revealed indirect effect of negative temperament axes on self-confidence through general imagery. To verify this hypothesis, the mediation moderated by discrete variable procedure described by Hayes and Preacher was applied.

Results of the analysis discovered that the latent variable of temperament axes formulation is similar in both athletes’ samples but regression weights in the model structure are different in both samples [Dc^2^(8) = 12.913; *p* = 0.115], which is presented in comparisons of constrained models presented in the Table 4 below.

**TABLE 3:** Summary of path coefficients.

		Path coefficients			Significance	
		b	SE	b	Z	p
**Latent variable formulation**						
Negative axes = ∼	Fear	0.91	0.11	0.63***	8.022	<0.001
	Anger	1.70	0.19	0.62***	8.743	<0.001
	Dissatisfaction	1.22	0.09	0.99***	14.963	<0.001
Positive axes = ∼	Sociability	5.38	1.32	0.68***	4.076	<0.001
	Activity	0.66	0.27	0.27*	2.444	0.015
**Regressions**						
Self-confidence ∼	Negative axes	−6.74	2.76	−0.26*	−2.442	0.015
	Positive axes	−0.96	3.88	−0.04	−0.247	0.805
	General imagery	0.16	0.08	0.22*	2.000	0.046
General imagery	Negative axes	−12.08	5.28	−0.33*	2.288	0.022
	Positive axes	24.71	7.38	0.37**	3.348	0.001
**Covariances**						
Negative axes ∼∼	Positive axes	−0.73	0.38	−0.73*^A^*	−1.921	0.055

^A^p < 0.1, *p < 0.05, ** p < 0.01, ***p < 0.001.

**TABLE 4:** Summary of model comparison for tested models with different restrictions constrained.

	Global fit indices							Difference from default model		
	c2	df	p	RMSEA	CFI	TLI	SRMR	c2	df	p
Default (non-constrained) model	13.121	10	0.217	0.059	0.937	0.906	0.093			
Restricted factor weights equality	18.196	14	0.198	0.075	0.922	0.866	0.096	5.075	4	0.280
Restricted regression weights equality	26.033	18	0.099	0.093	0.889	0.854	0.136	12.913	8	0.115
Restricted factor and regression weights equality	33.756	22	0.052	0.103	0.831	0.799	0.148	20.635	12	0.056

**FIGURE 2:**
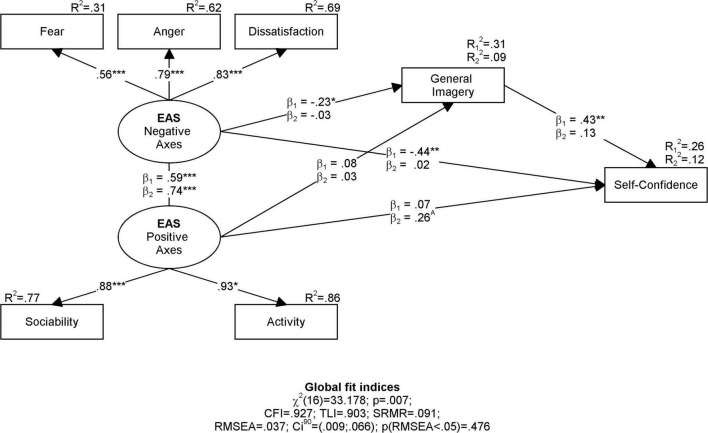
Result diagram of SEM model estimated in athletes training individual (*N*_1_ = 73) and team (*N*_2_ = 68) sports. Individual sports are coded as Group 1 and Team sports are coded as Group 2. *p < 0.05, **p < 0.01, ***p < 0.001.

Analysis of indirect effects conducted in the equal factors model revealed significant mediation of the general imagery in relation to negative temperament axes with self-confidence (*b*_*1*_ = –0.45; *p* < 0.01; *R*^2^_Med_ = 0.55) in individual sports sample, but an analogous effect was non-significant in team sports sample (*b*_*2*_ = –0.05; *p* = 0.371; *R*^2^ = 0.02). It suggests that general imagery explains the relation between negative temperament axes and self-confidence but only in individual sports athletes. The results are shown in the (Figure 2).

Detailed analysis of model obtained in both Individual and Teams Sports samples (Table 5) revealed that self-confidence is strongly aligned with negative axes of temperament in individual sport athletes (*b* = –0.44; *p* = 0.007), but analogous relation is rather trivial in team sports athletes sample (*b* = –0.02; *p* = 0.647). But the relation between self-confidence and positive axes of temperament is smaller in the sample of individual sport athletes (*b* = 0.07; *p* = 0.877) than team sport athletes (*b* = 0.26; *p* = 0.090). Self-confidence is either highly related to general imagery in individual sport athletes (*b* = 0.43; *p* = 0.004), but analogous relation in team sport athletes is trivial and non-significant (*b* = 0.13; *p* = 0.100).

These results suggest that general imagery explains the mechanism of being self-confident athlete in individual sport disciplines but is not a sufficient mediator in team sport disciplines.

**TABLE 5:** Summary of model comparison for tested models in individual and team sample.

		Individual sports (Group 1)					Team sports (Group 2)				
		b	SE	b	Z	p	b	SE	b	Z	p
**Latent variables (equal in both samples)**											
Negative axes = ∼	Fear	0.87	0.23	0.56	3.783	0.000					
	Anger	0.91	0.29	0.79	3.145	0.002					
	Dissatisfaction	1.03	0.22	0.83	4.682	0.000					
Positive axes = ∼	Sociability	0.99	0.19	0.88	5.254	0.000					
	Activity	0.76	0.21	0.93	3.621	0.000					
**Regressions**											
Self-confidence ∼	Negative axes	−0.51	0.19	0.44	−2.684	0.007	−0.11	0.24	−0.02	−0.458	0.647
	Positive axes	0.03	0.22	0.07	0.155	0.877	0.39	0.23	0.26	1.696	0.090
	General imagery	0.66	0.23	0.43	2.887	0.004	0.23	0.14	0.13	1.643	0.100
General imagery	Negative axes	−0.48	0.22	−0.23	−2.164	0.030	0.09	0.19	0.03	0.474	0.636
	Positive axes	−0.19	0.14	−0.08	−1.357	0.175	0.11	0.22	0.03	0.500	0.617
**Covariances**											
Negative axes ∼∼	Positive axes	−0.96	0.17	−0.58	−5.647	0.000	0.73	0.20	0.73	3.650	0.000

## Discussion

The main purpose of our study was to investigate the relationship among temperamental traits, a general tendency to use imagery, and self-confidence in sport. The specific aim was to verify if general imagery mediates the relationship between temperament and self-confidence in sport but also with respect to individual and team sport disciplines.

The results of the analysis concerning the first hypothesis that general imagery measured by ISQ is the mediator between temperament and self-confidence in the athletes’ population were partially supported. However, we discovered that general imagery was a significant mediator between only the emotionality (negative temperamental axes) and self-confidence and explained 46% of the variance. As far as positive temperamental axes were concerned, general imagery explained only 3% of self-confidence variance and it was insignificant.

Emotionality has the direct effect of reducing self-confidence but also on general imagery ability. Moreover, emotionality negatively affects self-confidence because it probably diminishes the general usage of imagery, which could be a tool for building self-confidence (Cumming and Williams, 2013; Munroe-Chandler et al., 2013; Cumming and Eaves, 2018). Positive axes support imagery ability in the analyzed population irrespectively of the team and individual discipline but this effect is limited by the type of sports discipline.

The results indicate that positive temperament traits do not directly promote self-confidence but directly promote general imagery, which in turn influences positively self-confidence. Additionally, general imagery does not mediate the relationship between the positive axes of temperament and self-confidence and in this sense does not explain why positive temperamental traits have no effect on confidence. This path is independent and other factors may influence this relationship more. If athletes have positive temperamental traits, then they use imagery positively and also present more often self-confidence. In the presented study, we analyzed only general imagery, which makes this study partially different from the previous research (Cumming and Williams, 2013; Munroe-Chandler et al., 2013; Cumming and Eaves, 2018).

Williams and Cumming (2012, 2016) revealed that mastery imagery ability was the strongest predictor of confidence, but also challenge and threat appraisals and cognitive anxiety. Both studies revealed that goal imagery ability positively predicts confidence. Similarly, mastery imagery was a mediator between a more specific type of confidence and performance (Beauchamp et al., 2002). The study of Quinton et al. (2017) discovered that positive and negative mastery imagery abilities mediated the relationship between confidence and challenge and threat appraisals but also the relationship between confidence and cognitive anxiety intensity. In positive mastery scripts, the mastery imagery ability was positively associated with confidence (Quinton et al., 2019). All studies concerned a mixed population of sport athletes (individual and team) and were connected to specific forms of imagery ability not the general one.

As far as team vs. individual sports are concerned and the second hypothesis, it turned out that the pattern from the whole group is similar only for individual sports. However, for team sports, we discovered a marginally significant positive direct effect between positive temperamental axes and self-confidence. This result means that higher levels of sociability and activity support self-confidence in team sports. This can be explained by the fact that team sports require the ability to cooperate and only people with such abilities train for this kind of sport thus self-confidence increases. Self-confidence in athletes can be strengthened by interacting with peers, presenting higher levels of confidence (Choi, 2017). It is also possible that players who differ in temperamental traits make up for certain deficiencies through the interpersonal relationships between team members. Moreover, team players had higher scores in extraversion (Malinauskas et al., 2014), conscientiousness, and autonomy than individual athletes, who have an advantage in agreeableness and sociotropy (Nia and Besharat, 2010).

Neither positive nor negative dimensions of temperament influence general imagery in the analyzed group of team sport. We also did not discover any relationship between general imagery and self-confidence. This is rather contradictory to previous studies, but they are mainly connected to the imagery intervention in team athletes (Heydari et al., 2018) or imagery use (Callow and Hardy, 2001) rather than imagery ability. In the study of Munroe-Chandler et al. (2013), imagery increases achieving goals of the performance not only in the individual but also in a team sport. In our study concerning self-confidence, we observed such a tendency only in an individual sport, which may be in accordance with the study of Callow et al. (2001), where motivational general-mastery ability (MG-M) was related to increased sport-confidence in badminton.

Our study is not free from limitations. The main weakness is the relatively small number of respondents, and we are aware of the rather pilot character of the study. Other traits connected with individual differences, which we did not measure, can affect the imagery ability, but also can differentiate imagery ability, i.e., mental toughness or other temperamental features measured by other questionnaires. They should be investigated in future research. In our study, we used only general imagery, but in the future, other measures of imagery ability should be included either. In our study, we verify our models for individual and team sports but it would be advisable to validate them with respect to gender and experience. We included basic information in the Supplementary Material. An in-depth analysis of other factors besides the nature of the discipline itself is justified. In future research, it is certainly valuable to explore further variables that could provide meaningful research insights.

The strength of our study and valuable insight is distinguishing the dependence of personal characteristics on imagery skills. Our study also extends the knowledge concerning aspects of “who” from the revised applied model of deliberate imagery use (RAMDIU, Cumming and Williams, 2013), which should be taken into account during cooperation with individual athletes. The other strength of our study was that our model was tested for both individual and team sports. Previous studies were connected to all kinds of sports disciplines (Quinton et al., 2017). Our models revealed significant paths which may be explained by the different roles of respondents’ imagery ability in the relation to temperament and self-confidence but also different sources of self-confidence depending on the type of their sports discipline (Malinauskas et al., 2014; Choi, 2017). It has been discovered that athletes in close or open sports, as well as contact and no-contact sports (Di Corrado et al., 2019), have varying levels of imagery ability. There is a possibility that the discipline diversifies the imagery ability in conjunction with confidence. Therefore, future research should focus on the sources and ways of confidence in team sports.

Our study also has practical implications, providing new concepts for imagery training. Working with general imagery ability as a feature (not only sport imagery) used in daily life may overcome temperamental difficulties associated with negative axes. However, it should be verified in the longitudinal study. Coaches and sport psychologists should be aware that athletes from individual disciplines who present higher emotionality would not be fluent in imagery ability, which in turn could be a tool for self-confidence. In conclusion, general imagery measured by ISQ is the mediator between emotionality and self-confidence in athletes. In terms of individual vs. team sports, we discovered that imagery ability plays a different role in relation to temperament and self-confidence. The spotlight on general imagery ability as the mediator between temperamental traits and self-confidence is a valuable insight into the research of imagery in sport.

## Data Availability Statement

The raw data supporting the conclusions of this article will be made available by the authors, without undue reservation.

## Ethics Statement

The studies involving human participants were reviewed and approved by the Ethics Committee of University of Gdańsk (protocol 11/2015). The patients/participants provided their written informed consent to participate in this study.

## Author Contributions

DB-P designed the study of the project, contributed to the acquisition, analysis, and interpretation of data as well as wrote the original draft of the manuscript. IH contributed to the interpretation of data and drafted the manuscript. KK contributed to the analysis, interpretation of data, drafted the manuscript, and concept and design of the project. All authors contributed to the revision of the manuscript and approved the final version for submission.

## Conflict of Interest

The authors declare that the research was conducted in the absence of any commercial or financial relationships that could be construed as a potential conflict of interest.

## Publisher’s Note

All claims expressed in this article are solely those of the authors and do not necessarily represent those of their affiliated organizations, or those of the publisher, the editors and the reviewers. Any product that may be evaluated in this article, or claim that may be made by its manufacturer, is not guaranteed or endorsed by the publisher.
