# Supplementary material for: Does Imagery Ability Matter for the Relationship Between Temperament and Self-Confidence in Team and Individual Sport Disciplines?
Source: Front Psychol. 2022 Jun 29;13:893457. doi: 10.3389/fpsyg.2022.893457 (PMC9311684; doi:10.3389/fpsyg.2022.893457)
Supplement: Supplementary file 1 [file Data_Sheet_1.docx]

#### Table 1:

*Correlation Matrix Between Age, Experience and ISQ Factors*

| **Variables** | | **Pearson's r** | **Benjamini-Yekutieli**  **95% Confidence Intervals** | | |
| --- | --- | --- | --- | --- | --- |
|  |  |  | Lower  Bound | Upper  Bound | p |
| *Age* | *Experience* | 0.239 | -0.049 | 0.491 | 0.004 |
|  | *Physiological feelings* | 0.037 | -0.249 | 0.317 | 0.660 |
|  | *Modalities* | 0.166 | -0.123 | 0.429 | 0.047 |
|  | *Ease/Control* | 0.146 | -0.143 | 0.412 | 0.081 |
|  | *Perspective* | 0.019 | -0.266 | 0.300 | 0.824 |
|  | *Affirmations* | 0.191 | -0.098 | 0.449 | 0.022 |
|  | *Visual* | 0.066 | -0.221 | 0.343 | 0.430 |
|  | *General* | 0.139 | -0.150 | 0.406 | 0.096 |
|  | *Dissatisfaction* | -0.162 | -0.425 | 0.127 | 0.053 |
|  | *Fear* | -0.177 | -0.438 | 0.111 | 0.033 |
|  | *Anger* | -0.139 | -0.406 | 0.150 | 0.097 |
|  | *Activity* | 0.152 | -0.137 | 0.417 | 0.069 |
|  | *Sociability* | 0.118 | -0.171 | 0.388 | 0.160 |
| *Experience* | *Physiological feelings* | 0.073 | -0.217 | 0.350 | 0.391 |
|  | *Modalities* | 0.055 | -0.234 | 0.335 | 0.516 |
|  | *Ease/Control* | 0.138 | -0.153 | 0.407 | 0.102 |
|  | *Perspective* | 0.003 | -0.282 | 0.288 | 0.974 |
|  | *Affirmations* | 0.186 | -0.105 | 0.447 | 0.027 |
|  | *Visual* | 0.076 | -0.214 | 0.353 | 0.372 |
|  | *General* | 0.093 | -0.197 | 0.368 | 0.272 |
|  | *Dissatisfaction* | 0.021 | -0.266 | 0.304 | 0.808 |
|  | *Fear* | -0.058 | -0.338 | 0.230 | 0.489 |
|  | *Anger* | -0.031 | -0.313 | 0.256 | 0.714 |
|  | *Activity* | -0.009 | -0.293 | 0.277 | 0.916 |
|  | *Sociability* | 0.144 | -0.147 | 0.412 | 0.087 |
| *Phys. feelings* | *Modalities* | 0.640 | 0.436 | 0.782 | < 0.001 |
|  | *Ease/Control* | 0.516 | 0.273 | 0.697 | < 0.001 |
|  | *Perspective* | 0.387 | 0.116 | 0.604 | < 0.001 |
|  | *Affirmations* | 0.509 | 0.265 | 0.693 | < 0.001 |
|  | *Visual* | 0.545 | 0.309 | 0.717 | < 0.001 |
|  | *General* | 0.293 | 0.011 | 0.532 | < 0.001 |
|  | *Dissatisfaction* | -0.050 | -0.328 | 0.237 | 0.554 |
|  | *Fear* | 0.165 | -0.123 | 0.428 | 0.048 |
|  | *Anger* | -0.103 | -0.375 | 0.185 | 0.218 |
|  | *Activity* | 0.158 | -0.131 | 0.422 | 0.058 |
|  | *Sociability* | 0.009 | -0.275 | 0.291 | 0.915 |
| *Modalities* | *Ease/Control* | 0.463 | 0.207 | 0.659 | < 0.001 |
|  | *Perspective* | 0.363 | 0.089 | 0.586 | < 0.001 |
|  | *Affirmations* | 0.450 | 0.191 | 0.650 | < 0.001 |
|  | *Visual* | 0.505 | 0.259 | 0.689 | < 0.001 |
|  | *General* | 0.247 | -0.039 | 0.495 | 0.003 |
|  | *Dissatisfaction* | -0.100 | -0.373 | 0.188 | 0.232 |
|  | *Fear* | 0.073 | -0.214 | 0.349 | 0.382 |
|  | *Anger* | -0.085 | -0.360 | 0.202 | 0.308 |
|  | *Activity* | 0.144 | -0.144 | 0.411 | 0.084 |
|  | *Sociability* | 0.069 | -0.218 | 0.345 | 0.411 |
| *Ease/Control* | *Perspective* | 0.783 | 0.642 | 0.872 | < 0.001 |
|  | *Affirmations* | 0.799 | 0.667 | 0.883 | < 0.001 |
|  | *Visual* | 0.704 | 0.526 | 0.823 | < 0.001 |
|  | *General* | 0.458 | 0.201 | 0.656 | < 0.001 |
|  | *Dissatisfaction* | -0.097 | -0.370 | 0.191 | 0.248 |
|  | *Fear* | -0.077 | -0.352 | 0.211 | 0.360 |
|  | *Anger* | -0.005 | -0.288 | 0.278 | 0.950 |
|  | *Activity* | 0.227 | -0.060 | 0.479 | 0.006 |
|  | *Sociability* | 0.081 | -0.206 | 0.356 | 0.332 |
| *Perspective* | *Affirmations* | 0.641 | 0.437 | 0.782 | < 0.001 |
|  | *Visual* | 0.575 | 0.348 | 0.738 | < 0.001 |
|  | *General* | 0.340 | 0.063 | 0.568 | < 0.001 |
|  | *Dissatisfaction* | -0.093 | -0.366 | 0.195 | 0.268 |
|  | *Fear* | -0.064 | -0.341 | 0.223 | 0.446 |
|  | *Anger* | -0.025 | -0.306 | 0.260 | 0.766 |
|  | *Activity* | 0.207 | -0.081 | 0.463 | 0.013 |
|  | *Sociability* | 0.063 | -0.224 | 0.340 | 0.452 |
| *Visual* | *Visual* | 0.667 | 0.474 | 0.799 | < 0.001 |
|  | *General* | 0.462 | 0.206 | 0.659 | < 0.001 |
|  | *Dissatisfaction* | -0.192 | -0.451 | 0.096 | 0.021 |
|  | *Fear* | -0.059 | -0.336 | 0.228 | 0.483 |
|  | *Anger* | -0.076 | -0.351 | 0.212 | 0.367 |
|  | *Activity* | 0.245 | -0.041 | 0.494 | 0.003 |
|  | *Sociability* | 0.210 | -0.078 | 0.465 | 0.012 |
| *Visual* | *General* | 0.376 | 0.103 | 0.595 | < 0.001 |
|  | *Dissatisfaction* | -0.126 | -0.395 | 0.163 | 0.134 |
|  | *Fear* | -0.039 | -0.318 | 0.247 | 0.644 |
|  | *Anger* | -0.070 | -0.346 | 0.218 | 0.406 |
|  | *Activity* | 0.169 | -0.119 | 0.432 | 0.042 |
|  | *Sociability* | 0.088 | -0.200 | 0.362 | 0.292 |
| *General* | *Dissatisfaction* | -0.254 | -0.501 | 0.032 | 0.002 |
|  | *Fear* | -0.165 | -0.428 | 0.124 | 0.048 |
|  | *Anger* | -0.132 | -0.400 | 0.157 | 0.116 |
|  | *Activity* | 0.262 | -0.023 | 0.507 | 0.002 |
|  | *Sociability* | 0.299 | 0.018 | 0.537 | < 0.001 |
| *Dissatisfaction* | *Fear* | 0.621 | 0.410 | 0.769 | < 0.001 |
|  | *Anger* | 0.616 | 0.403 | 0.765 | < 0.001 |
|  | *Activity* | -0.094 | -0.368 | 0.194 | 0.261 |
|  | *Sociability* | -0.474 | -0.667 | -0.220 | < 0.001 |
| *Fear* | *Anger* | 0.358 | 0.083 | 0.582 | < 0.001 |
|  | *Active* | -0.032 | -0.312 | 0.254 | 0.706 |
|  | *Sociability* | -0.295 | -0.533 | -0.013 | < 0.001 |
| *Anger* | *Activity* | 0.032 | -0.253 | 0.312 | 0.703 |
|  | *Sociability* | -0.313 | -0.547 | -0.033 | < 0.001 |
| *Activity* | *Sociability* | 0.155 | -0.134 | 0.419 | 0.064 |

####

#### Table 2:

*Descriptive Statistics of Temperament and ISQ Factors and Self Confidence in Sport in Males and Females Athletes*

| **Outcome**  **Variable** | **Females**  **(n = 58)** | | | | **Males**  **(n = 76)** | | | | **BY Corrected t** | | **Hedges g** | | |
| --- | --- | --- | --- | --- | --- | --- | --- | --- | --- | --- | --- | --- | --- |
|  | **M** | **SD** | **Skew** | **Kurt** | **M** | **SD** | **Skew** | **Kurt** | **t(132)** | **p** | **g** | **LB** | **UB** |
| *Age* | 20.59 | 1.60 | 0.37 | -1.13 | 20.51 | 1.76 | 0.98 | 0.46 | 0.633 | 0.551 | 0,11 | -0.17 | 0.33 |
| *Experience* | 6.65 | 3.93 | 0.35 | -0.70 | 8.08 | 4.40 | 0.31 | -0.93 | -4.559 | < 0.001 | -0,80 | -2.05 | -0.81 |
| *Sport Imagery* | | | | | | | | | | | | | |
| *Phys. feelings* | 21.56 | 6.05 | -0.67 | -0.10 | 20.18 | 6.32 | -1.10 | 0.86 | 2.981 | 0.002 | 0,52 | 0.46 | 2.30 |
| *Modalities* | 18.35 | 5.33 | 0.16 | -0.89 | 17.20 | 6.19 | -0.47 | 0.60 | 2.642 | 0.006 | 0,46 | 0.29 | 2.01 |
| *Ease/Control* | 39.57 | 6.27 | -0.73 | 0.52 | 39.46 | 9.91 | -2.43 | 0.76 | 0.173 | > 0.999 | 0,03 | -1.15 | 1.37 |
| *Perspective* | 29.04 | 6.53 | -0.40 | -0.69 | 28.59 | 8.59 | -1.43 | 0.50 | 0.776 | 0.367 | 0,14 | -0.70 | 1.60 |
| *Affirmations* | 33.00 | 5.04 | -0.77 | 0.14 | 32.50 | 2.10 | -2.49 | 0.48 | 1.825 | 0.051 | 0,32 | -0.04 | 1.04 |
| *Visual* | 23.10 | 4.40 | -0.44 | -0.73 | 23.13 | 5.73 | -2.55 | 0.95 | -0.077 | > 0.999 | -0,01 | -0.80 | 0.74 |
| *General* | 25.01 | 3.74 | -0.94 | 0.75 | 25.07 | 3.40 | -0.84 | 0.22 | -0.226 | 0.944 | -0,04 | -0.58 | 0.46 |
| *Temperamental Factors* | | | | | | | | | | | | | |
| *Dissatisfaction* | 10.60 | 3.27 | 0.36 | -0.36 | 10.76 | 3.30 | 0.10 | -1.08 | -0.652 | 0.485 | -0,11 | -0.65 | 0.33 |
| *Fear* | 10.49 | 2.99 | 0.11 | -0.56 | 8.72 | 2.62 | 0.14 | -0.40 | 8.515 | < 0.001 | 1,50 | 1.36 | 2.18 |
| *Anger* | 12.57 | 3.37 | -0.04 | -0.40 | 11.71 | 3.21 | 0.05 | -0.39 | 3.514 | < 0.001 | 0,62 | 0.38 | 1.34 |
| *Activity* | 14.37 | 3.03 | 0.11 | -1.10 | 13.41 | 2.77 | -0.24 | 0.07 | 4.459 | < 0.001 | 0,78 | 0.53 | 1.39 |
| *Sociability* | 13.63 | 3.16 | -0.29 | -0.41 | 13.63 | 2.96 | -0.26 | -0.41 | 0.000 | > 0.999 | 0,00 | -0.45 | 0.45 |
